# Perceived professional identity and related factors in Iranian nursing students: a cross-sectional study

**DOI:** 10.1186/s12912-022-01050-6

**Published:** 2022-10-13

**Authors:** Tahereh Gilvari, Hassan Babamohamadi, Fatemeh Paknazar

**Affiliations:** 1 Nursing Care Research Center, Semnan University of Medical Sciences, Semnan, Iran; 2 Department of Nursing, School of Nursing and Midwifery, Semnan University of Medical Sciences, 5 Kilometers of Damghan Road, Education and Research Campus, Po Box: 3513138111, Semnan, Iran; 3 Student Research commitee, University of Social Welfare and Rehabilitation Sciences, Tehran, Iran; 4 Social Determinants of Health Research Center, Semnan University of Medical Sciences, Semnan, Iran; 5 Department of Epidemiology and Biostatistics, School of Medicine, Semnan University of Medical Sciences, Semnan, Iran

**Keywords:** Professional, Identity, Nursing students, Iran

## Abstract

**Background:**

Professional identity, an important process in the development and expansion of the nursing profession, is built over time and includes gaining insight into professional performances and fostering ideals and values for the profession. Several factors influence the formation of professional identity. This study investigates the level of professional identity in nursing students and its predictors using a localized tool.

**Methods:**

This cross-sectional study recruited 195 nursing students at Semnan University of Medical Sciences, Semnan, Iran, who were selected by census sampling in 2020. Data were collected using a researcher-made professional identity questionnaire and were then analyzed in SPSS-18 using descriptive and inferential (logistic regression) statistics.

**Results:**

The mean total score of the students’ perceived professional identity was 316.72, indicating a strong professional identity. The students’ professional identity had a significant relationship with variables including GPA above 16 (OR = 2.65, P = 0.002), choosing the field out of interest (OR = 2.15, P = 0.015), and having work experience while studying (OR = 3.10, P = 0.006).

**Conclusion:**

The findings showed that selecting the field of nursing out of interest, having a GPA above 16 and work experience while studying are associated with a higher perception of professional identity among nursing students. The professional identity of nursing students can be enhanced through reinforcing the mentioned factors and further attention to their role in the promotion and consolidation of professional identity. The researchers recommend that educational directors, nursing professors, and clinical nursing educators make greater efforts to develop and promote the professional identity of nursing students.

## Background

Nursing shortages are a global concern, and the retention of nursing students merits greater attention from nursing scholars [1, 2]. Professional identity (PI) in nursing is defined as a “sense of oneself, and in relationship with others, which is influenced by characteristics, norms, and values of the nursing discipline, resulting in an individual thinking, acting and feeling like a nurse” [3].

The role of PI has been confirmed as a predictor of nurses’ retention [4, 5]. PI is a crucial part of the process of development and expansion of nursing as a profession [6]. This process takes place over time, involves gaining insight into professional performances and fostering professional ideals and values ​​[7], and is positively associated with students’ mental health, their clinical performance, patient satisfaction, and care quality [6, 8–10].

The status and prestige of any profession is related to the social identity of that profession. The sense of PI creates pride and satisfaction in the members of a group, enhances their participation in achieving the goals of the profession, creates solidarity among the members of the profession, and plays an important role in professional cohesion, unity and power [11, 12].

The formation of PI is an evolutionary process of maturity that begins with the start of the training course and never stops [13]. Nursing students form their PIs during the instructional process, which is conducted formally in the classroom and clinical wards, and covertly through informal experiences [14]. Clinical work by nursing students is crucial for developing their PI in the future [15, 16]. Commonly, people’s professional perception is formed in the first two to three years of entering a profession, and this perception, which forms the basis of the person’s PI, guides their professional actions throughout their career [17].

Having a positive PI is not only a factor that enhances the students’ self-confidence, sense of belonging to the profession and relationships with others, but also the most important factor in the development of job satisfaction and the best incentive for nurses to remain in the nursing profession [17]. The development of PI is one of the main concerns in nursing education and a major challenge in the development of professional roles in clinical settings [17]. Focusing on PI development is therefore essential for both clinical and nursing education arenas [18]. As a distinguishing feature of true nurses, nursing students can optimally achieve a strong PI through being exposed to and taking care of patients and empathetically meeting their needs [19].

## Introduction

Numerous factors can influence the formation of PI in students. Factors such as the existence of inconsistencies and differences between the nursing education environment and the clinical environment (i.e., the theory-practice gap) and the lack of a clear picture of the nursing profession in the society affect nurses’ PI [20]. Meanwhile, the students’ attitudes and ideals in relation to the nursing profession affect their motivation, choice of the nursing profession, and retention in the profession [21]. Studies suggest that students who select nursing as their first major of choice and study it with contentment have a better PI, and such a PI is strongly correlated with their continuing or leaving the profession [22].

Fairfield-Artman et al. (2010) reported that the process in which PI is formed in students is also influenced by their sexual identity, social networks, and relationships, especially with the university environment and their field of study [23]. The results of a study by Haghighat et al. (2020) showed that the score of PI is significantly higher in female students, married students, students with a higher GPA, and students who seems at is field with the nursing profession [24].

Studies emphasize the role of education as the key element in the formation of PI [25]. Preparing nursing students to achieve the desired professional competencies with an understanding of what is going on around them in the profession and having a clear vision of the future while taking into account the ongoing changes at the national and global levels requires some serious measures.

There is always a growing need for active professional nurses in hospitals, and the recruitment of capable students who are satisfied with their PI is a major concern of nursing schools [26], With this description, one of the duties of the officials of nursing schools is to be conscious of and to devise appropriate plans to improve the PI of nursing students [27].

To date, few studies have investigated PI and key factors affecting PI development in the Iranian society [25, 28]. Furthermore, PI depends on sociocultural context and not much research has been conducted on the sociocultural factors affecting the understanding of PI among nursing students in Eastern communities which have a different cultural background from Western communities [17]. Considering the importance of acquiring a PI and identifying the factors associated with the formation of a positive PI in students, the present study aimed at determining PI levels and its related factors in nursing students.

## Methods

### Design, setting and participants

This cross-sectional study was conducted from March 10th, 2020 to June 15th, 2020 to investigate PI and its predictors in nursing students at Semnan University of Medical Sciences.

The minimum required sample size for regression studies is 15–20 individuals per variable [29]. Since the present study had 12 main variables, the minimum sample size was calculated to be180; however, there were 210 eligible students. A total of 195 nursing students were selected and enrolled in the study by census sampling, and their data were ultimately analyzed.

The inclusion criteria were willingness and consent to participate in the study and being a nursing student in the 3rd semester or higher. Returning incomplete questionnaires and being a guest or a transfer student were taken as the exclusion criteria.

According to the Iranian undergraduate nursing education program, students enter clinical/hospital settings in the second semester. Given the development of PI in students from the 3rd semester onwards, being a student in the 3rd or higher semesters was considered an inclusion criterion of the study [22].

### Instruments and data collection

A PI questionnaire designed and psychometrically assessed in Iran and a researcher-made questionnaire on the factors affecting PI were used to collect the data.

The PI questionnaire has 63 items in six dimensions, including satisfaction with professional activity (items 1–23), professional commitment (items 24–35), professional transformation (items 36–46), personal growth (items 47–55), having a holistic view of the patient (items 56–59), and self-identifying as a nurse (items 60–63).The students scored themselves in each dimension of PI based on their experiences on a 6-point Likert scale (strongly disagree = 1, disagree = 2, somewhat disagree = 3, somewhat agree = 4, agree = 5, and strongly agree = 6). With this tool, the nursing students’ PI is obtained from the total points earned in each dimension. The minimum score is 63 and the maximum 378, and scores of 63–168 indicate poor PI, 169–273 moderate PI, and 274–378 strong PI. The closer the student’s score to 378, the higher their PI. The validity and reliability of this questionnaire have been assessed by Neishabouri (2017). The content and face validity of the questionnaire were approved by 15 faculty members based on CVR and CVI. The reliability of the questionnaire was assessed using the test-retest method in 30 nursing students on two occasions ten days apart. A reliability coefficient of more than 0.7 between the two tests was taken to indicate an acceptable stability. The reliability of the questionnaire was confirmed with Cronbach’s alpha coefficient of 0.964 and intraclass correlation coefficient (ICC) of 0.891 (P < 0.001). The reliability of the questionnaire in the stability assessment was equal to 0.88, which suggests the desirable stability of the PI assessment tool [30]. The reliability of the PI assessment tool was re-evaluated in the present study and confirmed by Cronbach’s alpha of 0.975, suggesting the high stability of the tool.

A researcher-made questionnaire based on a literature review was used to assess the factors affecting PI. The questionnaire consisted of 14 items on age, sex, marital status, place of residence, academic degree and semester, overall GPA, reason for choosing this field of study (including three items), being a guest or a transfer student, the rank of nursing among the student’s list of majors selected for the university entrance exam, nursing work experience and simultaneous work experience in the hospital as a student (student work) [21, 22, 31, 32].

### Ethical considerations

The research was approved by the Ethics Committee of Semnan University of Medical Sciences (Approval: IR.SEMUMS.REC.1398.250). The researchers started data collection after obtaining permission from the nursing school officials. The students who were eligible to enter the study were selected by taking a list of names from the faculty’s Office of Educational Services. The researchers visited the classrooms and hospitals (for senior students taking internships in the clinical setting) to collect the data. Since the current study was cross-sectional and the only risk was participants’ privacy, the researchers introduced themselves to the students and briefed them about the study objectives and methods, the confidentiality of the data, and the voluntary nature of participating in the study. The researchers answered students’ questions and informed consent for study participation was obtained from all nursing students.

The participants receiving the questionnaires in groups completed them individually in a classroom or a clinical setting. The questionnaires were completed in the presence of the authors and sampling was performed in a private room.

### Data analysis

Statistical analysis was performed using SPSS-18 software at a significance level of 0.05. The categorical variables were expressed as frequency and relative frequency and the quantitative ones as mean and standard deviation (SD). The Kolmogorov-Smirnov test was used to determine the normal distribution of the data before beginning their analysis. Since the normality assumption was not met for the PI scores in the present sample, the outcome variable was divided into two categories based on the median of 325 points, and a fitted multiple logistic regression model was then used to examine the relationship between each of the predictor variables and the recent two-state variable and estimating the odds ratio (OR). The final model was developed in three steps. First, the simple univariate models were fitted for each explanatory variable and the crude OR was obtained. A multiple model was then fitted in the presence of all the explanatory variables. In the third stage, a stepwise method and the likelihood ratio test were used to extract a reduced model from the multiple model. In these two steps, the adjusted OR was obtained for each variable and the main interpretation was performed based on the final model.

## Results

### Demographic characteristics of the participants

Of the 210 completed questionnaires, 15 had very high values of missing data and were thus excluded. The results showed that out of the 195 participants, 51.3% were female, 92.8% were single, and their mean age was 21.47 ± 2.41 years. Nursing major was among the first 15 choices of 48.7% of the participants after their success in the university entrance exam. Regarding the choice of the field of study, 67.7% entered the field with enough information, 73.3% for its future prospects, and 55.4% with enthusiasm. Also, 23% of the students worked while studying (Table 1).

**Table 1:** Demographic characteristics of the participants

	Frequency	Percentage
**Gender**		
Male	95	48.7
Female	100	51.3
**Marital status**		
Single	181	92.8
Married	14	7.2
**Place of residence**		
Urban	184	94.4
Rural	11	5.6
**Medical work experience before university admission**		
Yes	13	6.7
No	182	93.3
**What was the rank of nursing among your list of majors selected for the university entrance exam?**		
1–15	95	48.7
≥ 16	100	51.3
**Academic degree**		
Preclinical	79	40.5
Clinical	116	59.5
**Choosing the field with enough information**		
Yes	132	67.7
No	63	32.3
**Choosing the field with future prospects**		
Yes	143	73.3
No	52	26.7
**Choosing the field out of interest**		
Yes	108	55.4
No	87	44.6
**Simultaneous work experience in the hospital as a student (student work)**		
Yes	45	23.1
No	150	76.9
	**Mean**	**SD**
**Age**	21.47	2.41
**GPA**	15.76	1.17

### Distribution of mean scores of subscales and overall PI

According to the research findings, the mean score of the total PI from the students’ point of view was 316.72. Therefore, the PI of nursing students at Semnan University of Medical Sciences was categorized as strong. The highest score of PI was reported in the dimension of satisfaction with professional activity (115.53) and the lowest score in the dimensions of having a holistic view of the patient (20.39) and self-identifying as a nurse (20.88) (Table 2).

**Table 2:** Distribution of mean scores of subscales and overall PI

Subscales	Mean	SD	Min	Max
**Satisfaction with professional activity**	116.53	16.11	23	138
**Professional commitment**	57.62	10.21	12	72
**Professional transformation**	56.63	7.89	11	66
**Personal growth**	44.64	8.30	9	54
**Having a holistic view of the patient**	20.39	2.92	4	24
**Self-identifying as a nurse**	20.88	3.25	4	24
**Overall**	316.72	42.01	63	378

### The relationship between the predictor variables and PI in nursing students using the logistic regression models

Table 3 shows that PI had a significant relationship with the variables of GPA above 16 (OR = 2.65, P = 0.002), choosing the field out of interest (OR = 2.15, P = 0.015), and having work experience while studying (OR = 3.10, P = 0.006) in the students (Table 3).

**Table 3:** The relationship between the predictor variables and PI in nursing students using the logistic regression models

Variables	Univariate logistic regression model		Multiple logistic regression model		Backward stepwise multiple logistic regression model			
	OR	P-value	Adjusted OR	P-value	Adjusted OR	95% CI for OR		P-value
						Upper	Lower	
-Age above 20 years	2.08	0.020	1.32	0.478	-	-	-	-
-Female	1.25	0.423	-	-	-	-	-	-
-Married	3.71	0.050	2.31	0.263	-	-	-	-
-Rural	1.68	0.423	-	-	-	-	-	-
-Work experience before university admission	3.33	0.074	0.935	0.934	-	-	-	-
-Choosing the nursing field among the top 15 field	2.08	0.012	1.64	0.140	-	-	-	-
-Semester ≥ 6	2.58	0.002	0.972	0.946	-	-	-	-
-Choosing the field with enough information	2.28	0.009	1.07	0.886	-	-	-	-
-Choosing the field with future prospects	2.34	0.011	1.46	0.356	-	-	-	-
-Choosing the field out of interest	2.78	0.001	1.76	0.125	2.15	4.01	1.16	0.015
-Having work experience while studying	4.46	< 0.001	2.51	0.049	3.10	7.01	1.37	0.006
-GPA above 16	3.50	< 0.001	2.70	0.003	2.65	4.94	1.43	0.002

## Discussions

This study examined the PI of nursing students at Semnan University of Medical Sciences and its predictors. Based on the findings, the mean total score of PI was 316.72 in the students, which indicates their desirable PI.

In line with the present research, Skarbalius et al. (2018) examined the PI of nursing students in Lithuania and Poland using the Professional Identity Five-Factor Scale (PIFFS) and reported a high mean PI score of 4.03 ± 0.93 [33]. Mei et al. (2022) also reported a mean PI score of 84.82 ± 14.20 for nursing students [34]. Furthermore, a study by Chen et al.in 2020 reported a PI score of 83.22 ± 12.68 in Chinese undergraduate nursing students [35]. In contrast, in a study by Haghighat et al. (2020), the mean total score of PI of undergraduate nursing students was 55.61 ± 12.75 and categorized as moderate [24]. Also, in a study by Sun et al. (2016) on 623 undergraduate nursing students, the total score of PI was 57.63 ± 9.63, which is far from ideal [36]. All the cited studies used the PI scale (score = 17–85) developed by Hao et al. (2014) for nursing students [37]. The present study measured PI scores in the nursing students using a questionnaire with a score of 63–378 designed and psychometrically evaluated in a community of Iranian nursing students [30].

In justifying this disparity in results, it may be important to point out that the present study was conducted during the COVID-19 pandemic and the positive effects of mass media in showing the real status of nursing as a field of work and its being a specialized occupation should not be overlooked. Evidently, during this particular time, nursing students feel better about their chosen field because the efforts of nurses are being widely noticed, they receive constant appreciation from the community and there is a positive public feedback that could have affected their responses to the questionnaire items and generally their perception of PI.

Regarding the factors affecting the PI of nursing students, the results of the logistic regression showed a statistically significant relationship between the students’ GPA and PI score (P = 0.002). Consistently, the study by Heshmati et al. (2014) also showed that students with higher GPAs had higher PI scores (P = 0.003) [27]. Similarly, Haghighat et al. (2020) reported a significant positive relationship between GPA and the PI score (P = 0.002, r = 0.0211) [24, 31]. This finding can be explained by noting that in general, students with a higher and better perception of the field of nursing who attribute part of their identity to their chosen major tend to study harder, plan purposefully for their midterm and final exams, and consequently get better scores.

According to the present findings, a statistically significant relationship was observed between selecting the major out of interest and the PI score (P = 0.015). The students who had chosen nursing out of interest had higher PI scores. Consistently, the results of other studies show that students who have selected nursing as their first choice and who attend to it with contentment, enthusiasm and ample knowledge have a higher PI, and this PI is strongly associated with their retention in or leaving the profession [31, 32, 38]. Contrary to the present findings, in the study by Chen et al., only 27.6% of the students had chosen nursing out of interest [35]. The researchers attribute this contradiction to cultural differences, the students’ sexual identity and also the impact of social networks and students’ relationships, especially with the university environment.

The present findings showed a statistically significant relationship between work experience while studying nursing and the PI score (P = 0.006). In other words, students who worked in clinical settings while studying reported higher PI scores. Consistent with the present study, Chen et al. and Haghighat et al. showed that nursing students who worked in medical centers while studying had a higher PI (P = 0.001) [24, 35]. Hoeve et al. also showed that nurses can improve the public image of nursing and gain a stronger hold on healthcare organizations by increasing their physical presence in these settings; that is, nurses in strategic positions such as managerial or educator positions have the ability to demonstrate their field’s professionalism and its close tie with the society [39].

Research suggests that nursing students can develop an objective PI during clinical work and compare their perceptions with reality through familiarizing themselves with the work environment [22, 25].

A positive PI can be developed in nurses when nursing programs become focused on empowering PI and nursing managers create an atmosphere full of respect in the workplace [19, 40]. Therefore, it can be concluded that having work experience increases nursing students’ interactions with patients, families, staff and peers, and these interactions can determine their level of expertise and professionalism.

The present study found no statistically significant relationship between the PI of nursing students and their academic semester, while in the study by Haghighat et al. (2020), the students’ PI scores increased with their given semester (i.e., semesters 7 and 8) (P < 0.05) [24]. Sun et al. (2016) reported statistically significant relationships between semester and PI scores (r = 0.295, P < 0.01) [36]. To justify this difference, it can be argued that most students in Haghighat’s study were in the 7th and 8th semesters and had therefore gained various experiences while attending different clinical settings, including a combination of pleasant and unpleasant feelings, perceptions, understanding, and abilities. As a result, they had gradually gained the needed competency through their improved knowledge, attitude and skills, and found a more active role in the path of becoming professionals and had thus enhanced their PI [24]. Meanwhile, the samples in the present research consisted of a balanced combination of nursing students in different semesters.

Finally, the total score of the students’ PI had no statistically significant relationship with the variables of age, gender, marital status, place of residence, having relevant work experience before being admitted to the university, and selecting the field of nursing with previous knowledge and for its good future prospects. However, in the study by Sun et al. (2020), PI was significantly correlated with sex (P < 0.05) [41]. Nursing has traditionally been regarded as a female care-related occupation, and male nursing students and nurses had lower levels of PI than their female peers [32]. Moreover, in the study by Haghighat et al. (2020), the PI score was significantly higher in married women who had children and were satisfied with the nursing profession (P < 0.05) [24].

A study in China in 2016 showed that the total score of the PI of nursing students correlated with age (r = 0.145, P < 0.01), being the only child (r = 0.114, P < 0.05) and membership in social groups and committees (r = 0.151, P < 0.01) [36]. Chen et al. found that higher PI scores were reported by students living in rural areas, those continuing their scientific and educational activities during the holidays, those giving lectures at the university or hospital, and the students who had attended PI workshops in the university or hospital (P < 0.05) [35]. Zeng et al. (2022) reported that the mean PI score was higher in people who have a high school diploma, have siblings, reside in rural areas, studied their first-choice major, and had excellent scores at school [31]. Theresearchers believe that PI development is a process that is influenced by knowledge, professional authority, community approval, social culture, gender, work experience, teamwork skills, professional knowledge, interaction, and previous knowledge of the profession, and these factors may explain the disparities in the results obtained by Sun and Chen and those of the present study.

### Limitations of the study

Since this research enrolled only nursing students at Semnan University of Medical Sciences, its results should be cautiously generalized to other faculties and nursing education settings.

### Recommendations for future research

It is recommended that multicenter studies with larger samples be conducted in educational settings with different cultures to identify effective factors inPIin nursing students. Qualitative research is also required for explaining the process of PIdevelopment and comprehending the lived experiences of nursing students.

### Clinical implications for nursing managers and policymakers

Achieving a positive PI requires improving the nursing curricula based on reinforcing the professional position of nurses and improving their social status. A positive PI can be developed in nursing students based on the key role of nursing educators in creating a positive image of the profession and that of managers in creating a workplace atmosphere full of respect and promoting the image of nursing in clinical settings [17]. It is recommended that educational authorities, nursing professors, clinical nursing educators and policymakers develop strategies to promote PI in nursing students and present themselves as a flawless role model for future nurses.

## Conclusion

The results showed the desirable levels of perceived PI among the Iranian nursing students and factors such as selecting the field of nursing out of interest, GPA above 16, and work experience as a studentpositively affect perceivedPI among nursing students.

Acquiring knowledge on effective factors in PI development among nursing students is crucial for improving the teaching efficiency. Developing PI in nursing students can help turn them from a student to a professional nurse, increase their loyalty to the profession and promote the quality of care and patient satisfaction.

## Data Availability

All the data supporting the study findings are within the manuscript. Additional detailed information and raw data are available from thecorresponding author on reasonable request.

## Abbreviations

PI: Professional identity.
GPA: Grade point average.
OR: Odds ratio.
CVR: Content validity ratio.
CVI: Content validity index.
ICC: Intraclass correlation coefficient.
Min: Minimum.
Max: Maximum.
SD: Standard deviation.
